# Editorial: Bystander behavior in traditional bullying and cyberbullying: characteristics, antecedents, outcomes, and interventions

**DOI:** 10.3389/fpsyg.2024.1519007

**Published:** 2024-11-26

**Authors:** Xiaowei Chu, Nannan Zhou, Cuiying Fan, Marilyn Campbell

**Affiliations:** ^1^School of Psychology, Zhejiang Normal University, Jinhua, China; ^2^School of Psychology, Central China Normal University, Wuhan, China; ^3^School of Education, Queensland University of Technology, Brisbane, QLD, Australia

**Keywords:** bullying, cyberbullying, bystander behavior, types, predictors, mechanisms, interventions

## 1 Introduction

We know that bullying behavior occurs both in physical and virtual contexts and is associated with serious negative consequences. In bullying incidents, the role of bystanders is an important, although often overlooked one. Bystanders' positive or negative behavioral responses toward the bullying incident may alter its impacts and developmental course. However, until now, there is a scarcity of studies exploring bystanders' behavior in bullying, particularly in cyberbullying. To develop prevention or intervention strategies for bullying, this Research Topic provides an overview of original or review papers investigating bystander behavior in traditional bullying or cyberbullying in children, adolescents, and young adults.

The Research Topic consists of four original articles and one review article. The methodologies employed are both quantitative and qualitative analyses to explore specific questions concerning bystander behavior in bullying. Briefly, Rong et al. constructed a new classification model of bystander behavior in cyberbullying, Hu et al. examined the mechanisms in the relationship between empathy and bystanders' helping behavior in cyberbullying, Iotti et al. investigated the effects of autonomy-supportive parenting practices on adolescents' motivation to defend victims of bullying, Zhang et al. analyzed the mechanisms in the association between childhood psychological maltreatment and cyberbullying, and Cohane and Schneider evaluated the KiVa program as an effective anti-bullying program that highlights bystander intervention.

## 2 Main findings in this Research Topic

Rong et al. conducted two studies to analyze the structure of bystanders' behavior in cyberbullying. In Study 1, participants were presented with screenshots simulating a total of 32 cyberbullying scenarios and were asked to report their behavioral intentions to these scenarios. In Study 2, bystanders' actual behavioral responses were measured using a dynamic scenario simulation of eight cyberbullying incidents in a real online environment (i.e., QQ chat groups). Through these two studies, the results from qualitative analyses indicated that a proposed new classification model of bystander behavior in cyberbullying was generated. This model is a two-order factor structure, including three primary factors (i.e., positive, neutral, and negative bystander behavior) and six secondary factors (identifying the victim, bully, and others; inaction; supporting the bully and excessively confronting the bully).

Hu et al. investigated internet moral judgment and internet self-efficacy as the mechanisms accounting for the relationship between empathy and bystanders' helping behavior in cyberbullying. Participants completed questionnaires measuring these research variables. Results indicated that adolescents with stronger empathy were more likely to exhibit helping behavior in cyberbullying. In addition, internet moral judgment served as a mediator in this process, and internet self-efficacy moderated the latter half of the mediation path. That is, for participants with higher levels of internet self-efficacy, the association between internet moral judgment and helping behavior became much stronger.

Iotti et al. examined the role of autonomy-supportive parenting practices on young adolescents' self-reported motivation to defend victims of bullying, as well as the potential mediating roles of reactance, anxiety, depression, and stress. Researchers collected self-reported data from students aged 10–14 years from public schools in Italy. Results showed that autonomy-supportive parenting positively predicted autonomous defensive motivation to defend victims and negatively predicted extrinsic motivation to defend victims. These relationships were mediated by reactance. Specifically, autonomy-supportive parenting negatively predicted reactance, which further negatively predicted autonomous motivation to defend and positively predicted extrinsic motivation to defend.

Zhang et al. explored whether the relationship between childhood psychological maltreatment and cyberbullying perpetration was mediated by negative affect and moderated by meaning in life. Results revealed a significant positive correlation between childhood psychological maltreatment and cyberbullying. Additionally, negative affect partially mediated the relationship between childhood psychological maltreatment and cyberbullying. Meaning in life moderated the predictive effects of childhood psychological maltreatment and negative affect on cyberbullying, with these effects being stronger among individuals who reported lower levels of meaning in life.

Cohane and Schneider used a detailed narrative review to assess the capacity of the KiVa program to reduce bullying perpetration and victimization by examining randomized controlled trials conducted across different countries. This review suggests that KiVa is an effective intervention technique to reduce school bullying as well as to improve positive bystander responses to bullying. Although research has demonstrated the KiVa's effectiveness specifically at changing bystander behavior in some countries, such as Finland, Netherlands, and Chile, the cross-cultural effectiveness of this program needs further research.

## 3 Future directions

However, there are still many questions concerning bystander behavior in bullying that need to be explored. First, the existing classifications of bystanders' behavior in bullying are principally based on the nature or valence of specific actions (e.g., negative and positive). The classification standard is not specific and may be different in different bullying contexts. One essential criterion that distinguishes different types of bystanders' behavior might be the motivations to carry out specific acts. It has been shown that there are distinct motivations driving the same actions, particularly in the context of cyberbullying. For example, when witnessing a hurtful comment on social networking sites, actions such as “like” and “share” could indicate support for the victim of bullying but might indicate support for the perpetrator of the bullying. Future research could develop a motivation-based classification framework to clarify the complex nature underlying the same behavior.

Second, although many researchers have highlighted the important role that bystanders play in preventing bullying, this effect has not been empirically investigated. Future research might explore which types of bystanders' behavior prevent or effectively intervene in bullying. This requires a deeper investigation into the classification of bystander behavior. For one specific action, the intervention effect may be different across different contexts of bullying. For example, “doing nothing” may often be seen as a reinforcement of the bullies' behavior in school contexts. However, it might also be seen as a prosocial action, since “doing nothing” to some extent restrains the potential spread and negative effect of bullying incidents in cyber contexts.

Third, in traditional bullying and cyberbullying, the factors predicting bystander behavior and the mechanisms underlying these relationships can be compared. It seems with research so far, that personal and environmental factors play similar roles in affecting bystanders' behavior in both traditional bullying and cyberbullying. However, in cyberbullying, some predictive factors and mechanisms are unique and need to be highlighted. For example, on the one hand, anonymity may make bystanders more likely to confront the bullies as they fear less retaliation. On the other hand, anonymity may make bystanders less likely to intervene in bullying, since their expected reputation or social goals are weakened. Future research could examine the differences and commonalities in the factors and mechanisms predicting bystander behavior in both traditional bullying and cyberbullying.

## 4 Conclusion

In summary, this Research Topic offers an intriguing glimpse into the contemporary studies exploring the complex realm of bystander behavior in traditional and cyberbullying. These studies deepen our knowledge about the types, antecedents, mechanisms, and interventions for bystander behavior in these incidents. To provide more effective strategies for bullying prevention and intervention, future research needs to explore several critical aspects, such as the classification of bystanders' behavior based on other important criteria than just positive or negative, empirical investigation of bystanders' intervention effect, and comparison of factors predicting bystander behavior in different types of bullying.

